# A matched irrigation and obturation strategy for root canal therapy

**DOI:** 10.1038/s41598-021-83849-y

**Published:** 2021-02-25

**Authors:** Rafaela Fernandes Zancan, Mohammed Hadis, David Burgess, Zhenyu Jason Zhang, Alessandro Di Maio, Phillip Tomson, Marco Antonio Hungaro Duarte, Josette Camilleri

**Affiliations:** 1 Department of Restorative Dentistry, Dental Materials and Endodontics, Bauru School of Dentistry, University of São Paulo, Bauru, São Paulo Brazil; 2 School of Dentistry, Institute of Clinical Sciences, College of Medical and Dental Sciences, University of Birmingham, Birmingham, UK; 3 School of Chemical Engineering, University of Birmingham, Birmingham, UK; 4 School of Biosciences, University of Birmingham, Birmingham, UK

**Keywords:** Biomedical materials, Biomineralization, Biomaterials, Tissues, Medical research

## Abstract

In root canal therapy, irrigating solutions are employed to eliminate the bacterial load and also prepare dentin for sealer interaction. The aim of this research was to assess how irrigating solutions employed on their own or in sequence affected the tooth structure. The best way to prepare the tooth for obturation using hydraulic calcium silicate cement (HCSC) sealers and gutta-percha, thus guiding clinicians on a matched irrigation-obturation strategy for optimized root canal treatment was investigated. The effect of irrigating solutions on dentine was investigated by assessing changes in dentin microhardness, ultrastructure and mineral content, organic/inorganic matter, surface roughness and Young’s modulus. The interaction of four root canal sealers with the dentin was analysed by assessing the changes in microhardness of the dentin after sealer placement and also the sealer to dentin interface by scanning electron and confocal laser microscopy. The irrigating solutions damaged the dentin irreversibly both when used on their own and in combination. The best sequence involved sodium hypochlorite followed by chelator and a final rinse with sodium hypochlorite and obturation using HCSC sealers that enabled the restoration of dentin properties. The HCSC sealers did not rely on chelator irrigating solutions for a good material adaptation to dentin.

## Introduction

Root canal therapy is necessary following the loss of vitality of the dental pulp. The root canal is chemo-mechanically prepared to remove the pulpal remnants and associated bacteria^1^. The use of potent antimicrobial agents such as sodium hypochlorite (NaOCl) used in concentrations from 0.5 to 5% eliminates the microbial load^2^. The antimicrobial activity and efficacy of NaOCl for dissolving organic tissue, such as pulpal remnants from the canal space is concentration dependant^1,2^. Further degradation may lead to deleterious effects on the collagen matrix and integrity of the dentin structure^3^.

Mechanical instrumentation creates a smear layer which forms over the dentin surface blocking dentinal tubules. This smear layer can be removed by calcium chelating agents such as 17% EDTA which dissolve inorganic debris, however this subsequently reduces the mineral content of dentin causing peritubular and intertubular dentinal erosion^4^. As none of these irrigants can be regarded as optimal, they are used together to reduce the microbial load and also prepare the root canal dentin for the root canal obturation. The combined irrigant action enables a better outcome of the treatment in terms of cleaning and disinfection of root canal^5,6^. However, from a mechanical point of view, progressive dissolution of the organic and inorganic compounds of dentin may predispose to post-treatment root fracture resulting in tooth loss.

The chemo-mechanical preparation reduces the bacterial load and also leaves the dentine substrate ready to receive the obturating materials^6^. Traditionally root canal obturation is performed with gutta-percha and root canal sealers to hermetically seal the root canal. More recently the use of hydraulic calcium silicate cement (HCSC) sealers has resulted in a more dynamic obturation as such sealers exhibit antimicrobial properties^7,8^ and also interact with the dentin exhibiting bioactivity^9–11^. The interaction with the dentin is both chemical and micromechanical through sealer tags creating a self-adhesive bond with root canal walls^9–11^. The chemical bonds seem to have a positive effect on reinforcing the remaining tooth structure^12^. In this sense, sealers may cause recovery of the mechanical properties of dentin.

Since HCSC sealers interact with the dentin, it is important to determine the properties and the morphology of the latter after being subjected to the irrigating solutions used during root canal treatment. Therefore, the choice of sealer needs to be compatible with the irrigation protocol. Calcium chelation of dentin by EDTA is necessary for the sealer to bond to the exposed collagen and penetrate into the dentinal tubules after removal of the smear layer when using epoxy-resin-based sealers such as AH Plus (Dentsply-Sirona, Tulsa, OK, USA)^13^. HCSC sealers on the other hand do not benefit from the calcium chelation due to their particular chemistry^14^.

The aim of this research was to investigate a number of chemicals used as root canal irrigants and assess their effect on the dentin substrate. The best irrigation protocol leading to minimal damage to the dentin and resulting in the best substrate for interaction with HCSC sealers will be determined. This research also aims at guiding clinicians by prescribing the best irrigation protocol for root canal therapy using HCSC sealers for obturation.

## Results

The different irrigation protocols and sequences are shown in Table 1.

**Table 1:** Irrigants tested alone (NaOCl, EDTA, EDTA-BC and saline) or combined in different sequences.

Protocols	Groups	Irrigants
A	1	NaOCl 2%
	2	NaOCl 2%–EDTA
	3	NaOCl 2%–EDTA–NaOCl 2%
B	4	NaOCl 5%
	5	NaOCl 5%–EDTA
	6	NaOCl 5%–EDTA–NaOCl 5%
C	1	NaOCl 2%
	7	NaOCl 2%–EDTA-BC
D	4	NaOCl 5%
	8	NaOCl 5%–EDTA-BC
E	9	EDTA
F	10	EDTA-BC
G	11	Saline solution

*NaOCl* sodium hypochlorite, *EDTA* 17% ethylene diamine tetracetic acid, *BC* benzalconium chloride.

### Assessment of effect of irrigation protocol on dentin characteristics

#### Microhardness

The mean of Vickers hardness number (VHN) measurements of root dentin according to the treatment group in different protocols are shown in Table 2. The use of 2% concentration of NaOCl (G1; NaOCl-2; Cerkamed, Stalowa Wola, Poland; Protocols A and C) did not result in changes in dentin microhardness (p = 0.45 and p = 0.73). However, there was a significant reduction in dentin microhardness in protocols B and D when 5% NaOCl (G4; NaOCl-5; Cerkamed, Stalowa Wola, Poland) was used (p = 0.02; p = 0.002). 17% EDTA (G9; EDTA; Sigma, Stoinheim, Germany; Protocol E), 17% EDTA/1% benzalkonium chloride (EDTA-BC; Sigma, Stoinheim, Germany; Protocol F-G10) and saline (Protocol G-G11) used without the NaOCl did not affect the microhardness of dentin (p = 0.70; p = 0.10; p = 0.86 respectively). When EDTA was used following 5% NaOCl as in Protocol B, Group 5, the dentin microhardness was significantly reduced (p = 0.02). The inclusion of the 1% BC in EDTA counteracted the effects of the 5% NaOCl on Group 8 and no reduction in the dentin microhardness occurred in Protocol D (p > 0.05).

**Table 2:** Mean (X) and standard deviation (SD) values of Vickers hardness number (VHN), amide III/phosphate and carbonate/phosphate bands of dentin surface before (initial—I) and after immersion in the irrigation solutions (G1–G11) as indicated in protocols A–G which are detailed in Table 1.

Protocols	Irrigants	Groups	Vickers hardness before obturation	Vickers hardness after obturation	AmideIII/phosphate band	Carbonate/phosphate band	Young`s modulus
			VHN X (SD)	VHN Med (min–max)	Ratio X (SD)	Ratio X (SD)	MPa Med (min–max)
A		I	66.89^A^ (6.85)		52.72^A^ (55.25)	20.43^A^ (1.76)	535.5^A^ (143–1127)
	NaOCl 2	G1	65.13^A^ (4.71)	65.74^A^ (49.8–112)	27.16^A^ (25.74)	22.47^A^ (4.99)	353.5^AB^ (119–1968)
	NaOCl 2–EDTA	G2	60.45^A^ (3.28)	63.78^A^ (55.4–119)	100.9^A^ (118.7)	17.50^A^ (6.85)	5.94^C^ (2.27–11.60)
	NaOCl 2–EDTA–NaOCl2	G3	59.85^A^ (2.92)	67.70^A^ (57.2–90.7)	31.81^A^ (19.87)	22.12^A^ (3.15)	327^B^ (7.75–1652)
B		I	71.68^A^ (9.49)		156.1^A^ (302.5)	17.26^AB^ (1.85)	756^A^ (96.5–6564)
	NaOCl 5	G4	62.35^AB^ (0.62)	72.60^A^ (53.3–134)	18.06^A^ (9.78)	20.77^A^ (2.51)	173.5^B^ (63.30–967)
	NaOCl 5–EDTA	G5	48.76^BC^ (7.15)	66.90^A^ (39.1–116)	99.60^A^ (84.83)	14.26^B^ (5.23)	10.2^C^ (3.38–28.0)
	NaOCl 5–EDTA–NaOCl 5	G6	35.63^C^ (4.92)	61.65^A^ (35.8–100)	39.99^A^ (45.16)	19.15^AB^ (3.99)	615.5^A^ (81.3–3120)
C		I	73.94^A^ (9.49)		89.10^A^ (89.60)	21.32^A^ (5.26)	214.5^A^ (42.50–1135)
	NaOCl 2	G1	69.64^A^ (10.14)	65.74^A^ (49.8–112)	41.10^A^ (23.87)	26.03^A^ (6.36)	203.5^B^ (50.10–1110)
	NaOCl 2–EDTA-BC	G7	67.40^A^ (9.86)	69.20^A^ (32.4–114)	56.50^A^ (24.47)	19.35^A^ (5.02)	8.83^B^ (4.99–65.6)
D		I	85.43^A^ (3.86)		18.08^B^ (3.05)	18.49^A^ (3.59)	839.5^A^ (12.9–1933)
	NaOCl 5	G4	75.43^B^ (1.94)	72.60^A^ (53.3–134)	9.37^B^ (5.67)	24.08^A^ (4.42)	95.00^B^ (15.5–765)
	NaOCl 5–EDTA-BC	G8	69.96^B^ (3.44)	72.40^A^ (57.6–120)	52.28^A^ (30.18)	23.95^A^ (4.73)	19.40^C^ (9.13–68.70)
E		I	60.71^A^ (12.70)		41.38^A^ (35.37)	26.15^A^ (5.60)	364.5^A^ (36.8–1192)
	EDTA	G9	54.65^A^ (9.01)	72.70^A^ (48.9–101)	90.46^A^ (88.22)	16.67^A^ (3.33)	13.25^B^ (3.87–30.0)
F		I	64.15^A^ (6.33)		14.01^A^ (3.14)	22.46^A^ (3.17)	100^A^ (27.9–255.0)
	EDTA-BC	G10	47.88^A^ (1.45)	82.10^A^ (46.2–116)	59.65^A^ (48.43)	19.91^A^ (0.92)	11.9^B^ (5.63–84.10)
G		I	64.37^A^ (0.50)		17.41^A^ (3.96)	20.60^A^ (1.25)	501^A^ (94.3–1275)
	Saline solution	G11	64.30^A^ (0.36)	90.30^A^ (45.5–108)	16.83^A^ (4.06)	20.67^A^ (1.42)	285.0^B^ (118–597)

Median (Med), Maximum (Max) and Minimum (Min) values of Young`s modulus of coated dentin surface as indicated before and it Vickers hardness number after obturation.

#### Morphology and composition of dentin

##### Scanning electron microscopy (SEM) and energy dispersive spectroscopy (EDS)

**Figure 1:**
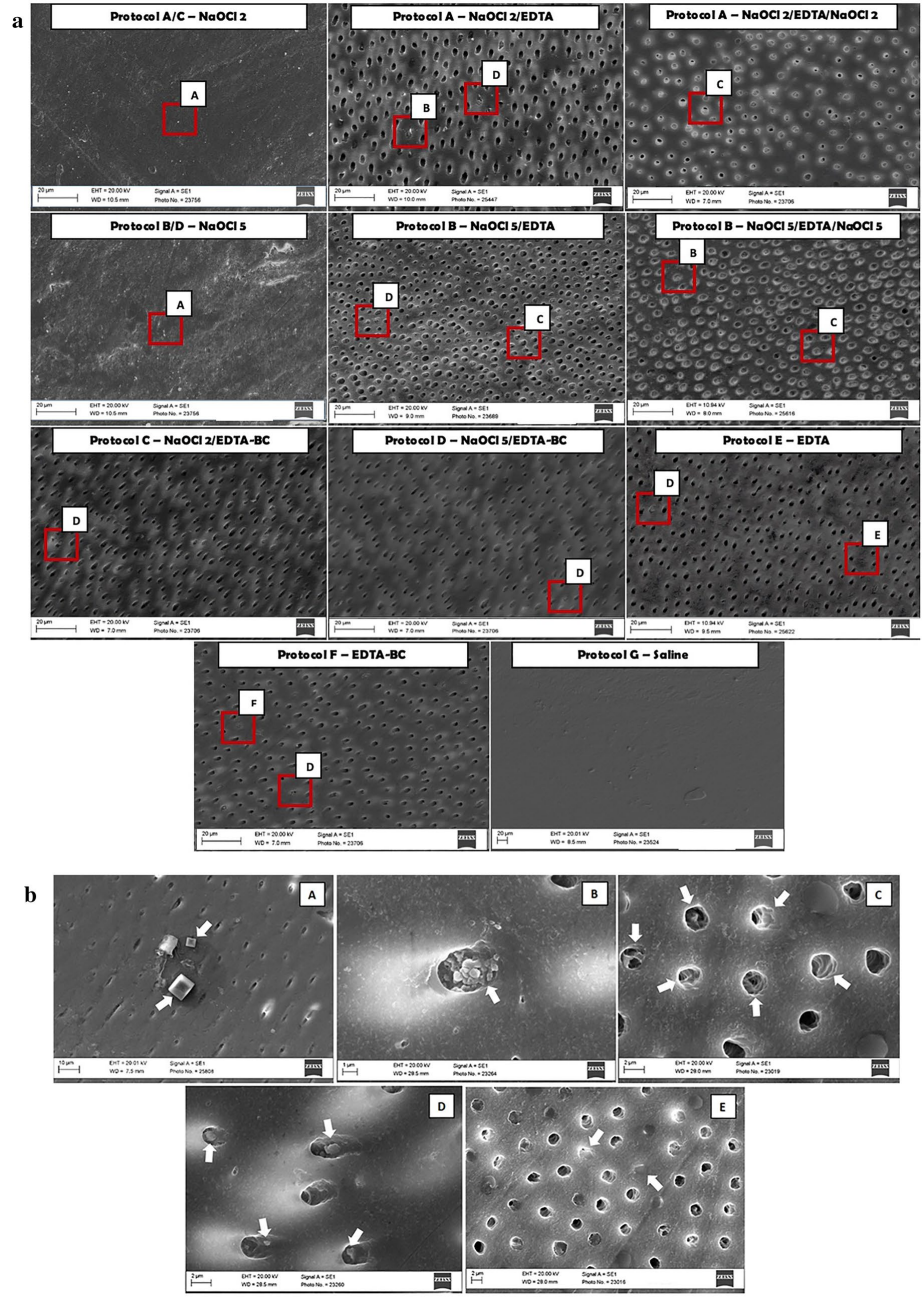
(**a**) Representative images of root dentin at × 2 K magnification after the different irrigation protocols on the surface. The letters on the images indicate the areas that were viewed at higher magnification and are shown in (**b**). (**b**) High magnification scanning electron micrographs (3 K ×: A,C,E; 4 K ×: B,D); of the areas marked by the letters A–E in Figure (**a**). The areas are indicated with white arrows. (A,B) shows the presence of cuboid crystals rich in sodium and chlorine on dentin and inside dentinal tubules, respectively; (C) shows the peritubular and intertubular dentinal erosion; (D) shows the presence of smear layer inside dentinal tubules and (E) shows the sclerosis of dentinal tubules.

The scanning electron micrographs of the dentin treated with different irrigation protocols are shown in Fig. 1a and higher power images of specific areas are shown in Fig. 1b. Both concentrations of NaOCl failed to remove the smear layer as is indicated in Protocols A and B, when the NaOCl was used as the first irrigant. Small deposits were observed on the dentin surface (marked A in Fig. 1a). High magnification of these deposits (Fig. 1b) revealed the cuboid shape of the crystals which is typical of sodium chloride^16^. This was confirmed by EDS analysis. For both Protocol A and B, the use of EDTA after the NaOCl, led to the widening of the dentinal tubules which was more effective in the 2% sodium hypochlorite group (Fig. 1a). After EDTA irrigation some crystals of sodium chloride were visible inside the tubules (area marked as B in Fig. 1a) in certain areas shown in high magnification in Fig. 1b. A final rinse with sodium hypochlorite resulted in the erosion of the dentin around the tubules (Fig. 1a area marked as C).

The use of EDTA-BC after the NaOCl as in Protocols C and D resulted in widening of the dentinal tubules with some erosion. The dentinal tubules were less evident than when using EDTA after the hypochlorite as in Protocols A and B. After the use of chelators small amounts of smear layer were present inside dentinal tubules for Protocols A, B, C and D (Area marked as D in Fig. 1a shown in high magnification in Fig. 1b), which disappeared after the final flush of sodium hypochlorite. The use of EDTA and EDTA-BC as sole irrigating solutions in protocols E and F showed widening of the dentinal tubules also with some sclerosis (Fig. 1a area marked E) at the periphery and presence of smear layer. Saline used on its own as in Protocol G did not have any effect on the smear layer removal.

Table 3 summaries the percentage of elemental composition for calcium, phosphate, chlorine and silicon in the EDS analysis of dentin surface. The use of saline (G11) and 5% NaOCl (G4) resulted in the highest values of phosphate and chlorine, respectively.

**Table 3:** Median, Minimum and Maximum values of the percentage of calcium (Ca), phosphate (P), chlorine (Cl) and silicon (Si) obtained from the Energy Dispersive Spectroscopy (EDS) chemical analysis on dentin surface after immersion in the irrigation solutions (G1–G11) as indicated in protocols A–G which are detailed in Table 1, before and after root canal obturation.

Dentine management	Element	G1	G2	G3	G4	G5	G6	G7	G8	G9	G10	G11
Before obturation	Ca	22.69^A^ (21.4–28.0)	18.07^A^ (17.5–23.3)	18.96^A^ (16.6–19.1)	25.79^A^ (18.7–26.1)	18.60^A^ (16.2–23.2)	17.69^A^ (13.9–24.1)	15.43^A^ (14.1–15.5)	16.05^A^ (12.5–18.3)	18.80^A^ (12.4–20.5)	16.42^A^ (12.4–20.1)	24.64^A^ (24.5–24.8)
After obturation	Ca	27.84^A^ (22.5–32.8)	26.43^A^ (19.5–30.3)	29.48^A^ (22.1–32.4)	25.52^A^ (22.8–28.7)	27.41^A^ (22.9–33.6)	27.50^A^ (23.1–30.3)	27.67^A^ (22.5–32.9)	27.36^A^ (19.9–31.2)	19.72^B^ (12.9–28.3)	20.67^B^ (16.3–27.6)	25.51^A^ (22.3–32.0)
Before obturation	P	12.35^AB^ (11.7–14.5)	10.18^AB^ (9.7–11.9)	10.70^AB^ (10.5–11.3)	13.80^AB^ (10.2–14.0)	9.81^AB^ (7.9–13.3)	9.94^AB^ (8.9–13.3)	8.57^AB^ (7.9–8.69)	4.99^B^ (3.19–10.0)	9.52^AB^ (7.81–11.2)	10.95^AB^ (5.5–11.4)	13.43^A^ (13.2–13.8)
After obturation	P	14.42^A^ (11.7–17.2)	14.03^AB^ (8.2–18.1)	13.85^AB^ (10.5–17.2)	12.56^A^ (10.8–14.4)	14.48^A^ (12.2–15.6)	14.05^AB^ (9.3–16.0)	13.74^AB^ (11–16.4)	14.07^AB^ (10.7–16.5)	10.71^C^ (8–15)	11.75^BC^ (9.-15.1)	12.76^AB^ (9–17.1)
Before obturation	Si	0.0^A^ (0.0–0.0)	0.0^A^ (0.0–0.0)	0.0^A^ (0.0–0.0)	0.0^A^ (0.0–0.0)	0.0^A^ (0.0–0.0)	0.0^A^ (0.0–0.0)	0.0^A^ (0.0–0.0)	0.0^A^ (0.0–0.0)	0.0^A^ (0.0–0.0)	0.0^A^ (0.0–0.0)	0.0^A^ (0.0–0.0)
After obturation	Si	0.0^A^ (0.0–0.0)	0.0^A^ (0.0–1.6)	0.0^A^ (0.0–0.21)	0.0^A^ (0.0–0.3)	0.0^A^ (0.0–0.3)	0.0^A^ (0.0–0.44)	0.0^A^ (0.0–0.09)	0.0^A^ (0.0–0.24)	0.0^A^ (0.0–0.0)	0.0^A^ (0.0–0.0)	0.0^A^ (0.0–0.0)
Before obturation	Cl	1.05^AB^ (0.97–2.35)	0.71^AB^ (0.26–0.79)	0.16^AB^ (0.11–0.79)	3.15^A^ (1.58–6.22)	1.08^AB^ (0.52–1.45)	0.20 ^AB^ (0.11–0.35)	1.02^AB^ (0.93–1.14)	1.06^AB^ (0.27–3.15)	0.0^B^ (0.0–0.0)	0.30^AB^ (0.05–1.43)	0.0^B^ (0.0–0.0)
After obturation	Cl	0.15^BCD^ (0.0–1.00)	0.25^ABC^ (0.13–0.58)	0.24^ABCD^ (0.0–1.47)	0.20^BCD^ (0.0–0.93)	0.16^BCD^ (0.04–0.75)	0.45^A^ (0.30–4.01)	0.28^AB^ (0.14–1.38)	0.28^ABCD^ (0.12–0.92)	0.0^CD^ (0.0–1.52)	0.15^BCD^ (0.0–0.51)	0.0^D^ (0.0–0.44)

Different capital letters in rows indicate statistically significant intragroup differences (Kruskal–Wallis with a Dunn post hoc P value < 0.05).

##### Fourier transform infrared spectroscopy (FT-IR)

**Figure 2:**
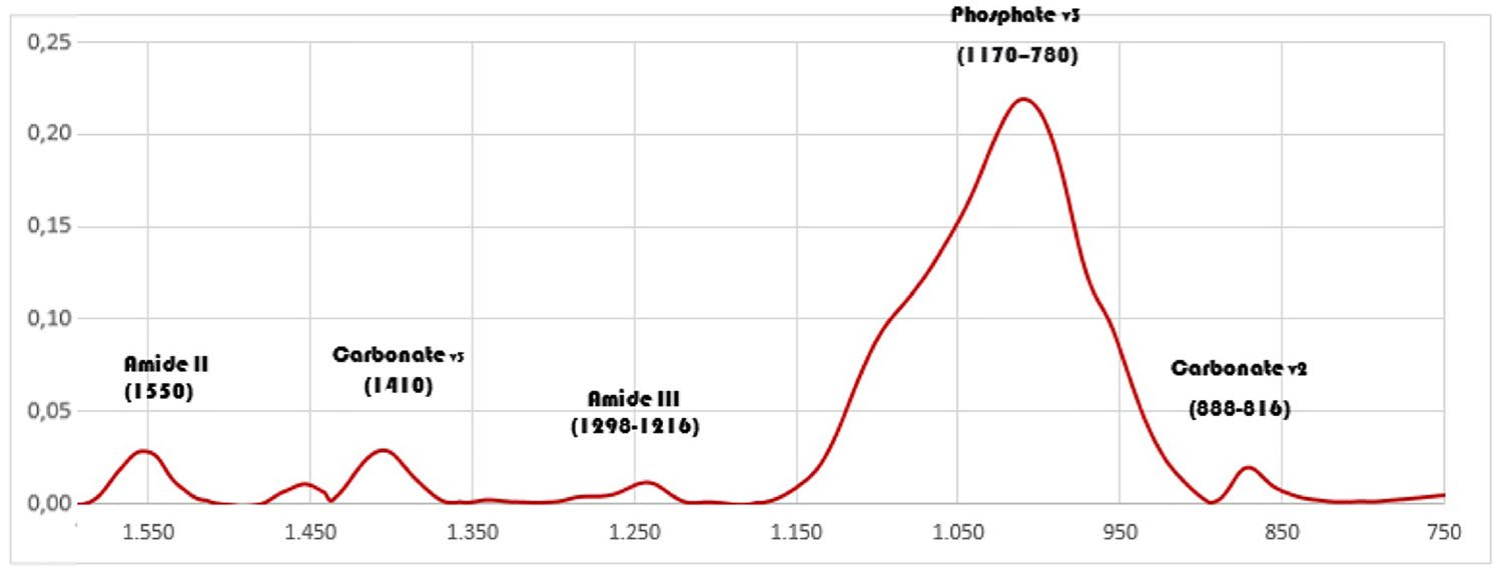
Representative infrared spectral region between 1600 and 750 cm^−1^ of untreated dentin showing the absorption peaks of the main dentin components.

The infrared spectral region between wavenumbers 1600 and 750 cm^−1^ of untreated dentin is shown in Fig. 2 indicating the absorption peaks amide III (1298–1216 cm^−1^), phosphate (1170–780 cm^−1^) and carbonate (888–816 cm^−1^). Table 2 shows values for the ratios of amide III/phosphate and phosphate/carbonate respectively, on the dentin surface before and after immersion in the irrigants contained in the Protocols A–G. NaOCl first (G1 and G4) and final flush (G3 and G6) caused a decrease on organic matter by the deproteinization of collagen on dentin that is recovered by the follow use of chelators, although these changes were not sufficient to alter significantly the amide III/phosphate ratio (Protocols A-G2, B-G5 and C-G7; Table 2). When NaOCl was followed by EDTA-BC (Protocol D-G8) the collagen ratio increased significantly (p = 0.004). The previous use of the higher concentrated NaOCl (Protocol B-G4) seems to stimulate the demineralization of dentin by EDTA (p = 0.015; Table 2; G5) in comparison to 2% NaOCl (p ˃ 0.05; Protocol A-G2).

##### Atomic force microscopy (AFM) analysis

**Figure 3:**
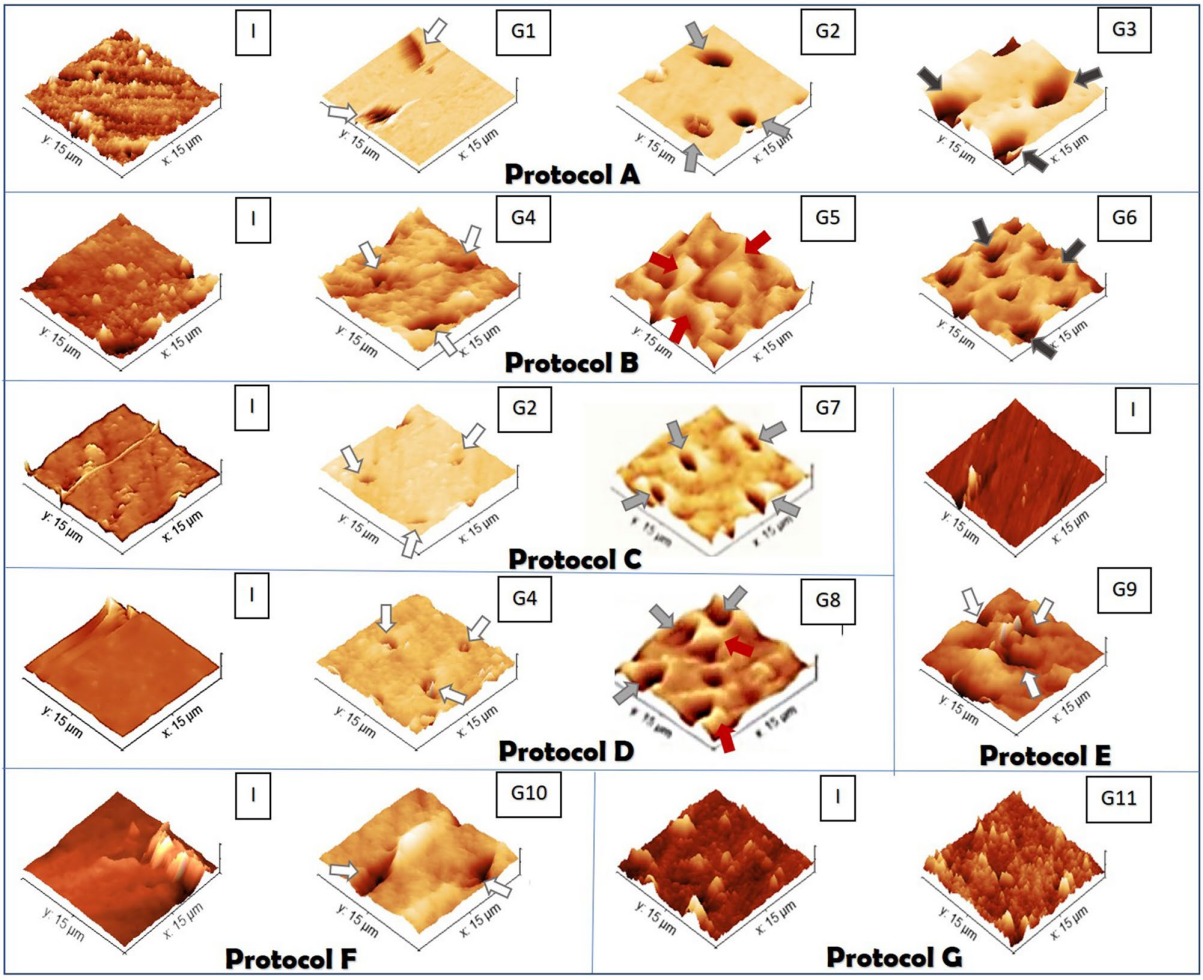
Atomic force micrographs showing topography of the dentin surface before (initial—I) and after immersion in the irrigation solutions (G1–G11; horizontal axis) as indicated in Protocols A–G detailed in Table 1. White arrows indicate less evident dentinal tubules in groups with NaOCl or chelator single flush. Light grey and dark grey arrows expressed a wider range and progressive widening of the dentinal tubules when both irrigants were used in a Protocol alone or adding NaOCl as a final flush, respectively. In protocol NaOCl+ chelator, (5%) NaOCl promoted a rough surface of dentin indicated by red arrows.

The Young’s modulus data is shown in Table 2 and representative AFM images (Fig. 3) show the changes to the dentin samples subjected to the irrigation protocols. Based on Table 2, it is clear that the Young’s modulus of the dentin was altered by NaOCl used as the first irrigating solution (G1 and G3), chelators such as EDTA (G9) used on its own and saline (G11). When combined in a protocol, the proteolytic effect of NaOCl on dentin collagen followed by the chelator demineralization effect on dentin caused a progressive decrease in dentin Young’s Modulus (Protocols A-G2, B-G5 and D-G8; p < 0.001). Interestingly, NaOCl final flush increased these values (Protocols A-G3 and B-G6; p < 0.001), to the original levels (p ˃ 0.05). The AFM images (Fig. 3) show the smear layer that totally covered the dentin in the initial step. The attenuation on image colors after the first application of NaOCl on dentin is related to a decrease of smear layer height on its topography. When the chelators were used alone as in Protocols E-G9 and F-G10, only a limited number of dentinal tubules could be observed. The irrigation protocols that included both the NaOCl and the EDTA resulted in a wider range of open dentinal tubules (G2 and G5; marked with light grey arrow), whereas the 5% NaOCl promoted a rougher dentin surface compared to the 2% concentration (marked with red arrow). A smoother surface and widening of the dentinal tubules (dark grey arrows) was observed after the final step of irrigating with NaOCl in Protocols A-G3 and B-G6.

### Assessment of the dentin to sealer interface

#### Microhardness testing

Table 2 shows the mean of Vickers hardness number (VHN) measurements of root dentin after obturation according to the treatment group in different irrigant protocols. No statistical differences were seen on the microhardness of dentin treated with different irrigant protocols followed by root canal obturation.

Table 4 shows the mean of Vickers hardness number (VHN) measurements of root dentin after obturation according to the sealer used. MTA Fillapex and Totalfill enhanced dentin microhardness indicating a recovery of the dentin previous affected by the different irrigation protocols.

#### Assessment of the dentin to sealer interface

##### Energy dispersive spectroscopy (EDS) in SEM

Tables 3 and 4 show the percentages of elemental composition for calcium, phosphate, chlorine and silicon in the EDS analysis of the dentin treated with different irrigation protocols followed by root canal obturation. The same data was used for both tables. In Table 3 the data was presented according to the irrigant protocol used, regardless of sealers. In Table 4 the data was presented according to the sealer used, regardless of the irrigation protocol.

**Table 4:** Median (Med). Minimum (Min) and Maximum (Max) values of the Vickers hardness number (VHN) of dentin surface after root canal obturation and it percentage of calcium, phosphate, chlorine and silicon obtained from the Energy Dispersive Spectroscopy (EDS) chemical analysis.

	AH plus Med (min–max)	BioRoot Med (min–max)	MTA Fillapex Med (min–max)	Total Fill BC sealer Med (min–max)	Non-obturated teeth Med (min–max)
VHN	62.10^B^ (49.80–108)	69.20^B^ (45.50–116)	89.30^A^ (55.40–120)	99.70^A^ (60.10–134)	61.65^B^ (32.40–77.80)
Calcium	25.44^A^ (12.94–32.01)	27.29^A^ (19.55–32.96)	26.41^A^ (15.07–33.63)	26.01^A^ (19.45–29.82)	18.35^B^ (2.74–28.04)
Phosphate	13.25^A^ (8.08–17.11)	13.37^A^ (8.80–18.14)	14.39^A^ (9.07–17.23)	12.5 ^A^ (9.07–15.62)	10.55^A^ (3.19–14.58)
Chlorine	0.16^B^ (0.0–0.58)	0.58^A^ (0.0–4.01)	0.21^B^ (0.0–0.92)	0.18^B^ (0.0–0.60)	0.62^A^ (0.0–6.22)
Silicon	0.0^C^ (0.0–0.0)	0.0^BC^ (0.0–0.27)	0.0^BC^ (0.0–1.64)	0.0^AB^ (0.0–0.44)	0.0^C^ (0.0–0.0)

Different capital letters in rows indicate statistically significant intragroup differences (Kruskal–Wallis with a Dunn post hoc P value < 0.05).

Table 3 shows that the lowest calcium and phosphate values were found for the chelator groups using EDTA (G9) and EDTA-BC (G10). The irrigation protocol NaOCl followed by EDTA and final NaOCl flush resulted in higher amounts of chlorine in Protocol B-G6, (p < 0.0001) when compared to NaOCl alone (G1 and G4) or followed by EDTA (G5), chelator groups (G9 and G10) and saline (G11). No statistical differences were seen among the irrigation protocols on the amounts of silicon on dentin (p > 0.05).

Table 4 shows that all sealers enhanced the calcium values of dentin (p < 0.0001). Chlorine remained on dentin after applying the irrigation protocols. BioRoot had the highest chlorine values among the sealers, due to the presence of this ion within its composition (p < 0.0001).

##### Confocal laser scanning microscopy (CLSM)

**Figure 4:**
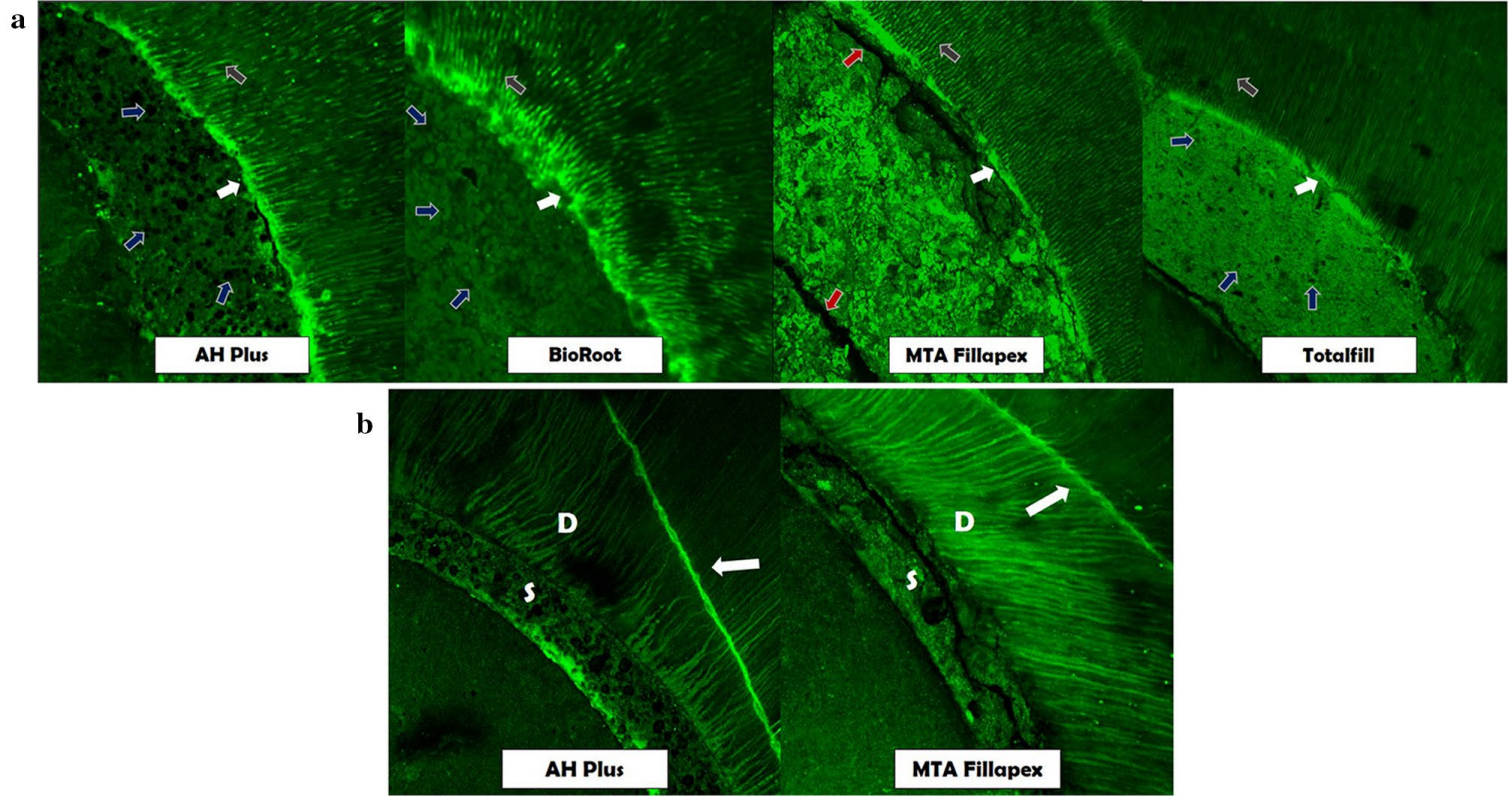
Representative fluorescence-mode CLSM images of sealer-dentin interface demonstrated by Orange G added to the test sealers; (**a**) showing a richly dye-infiltrated layer at the sealer-dentin interface marked with white arrows, sealer penetration inside dentinal tubules marked with grey arrows, porosity of sealers with blue arrows and cracks on sealer mass with red arrows; (**b**) cracks indicated by fluorescent radial lines on dentin (D) marked by white arrows and on the sealer (S) for MTA Fillapex indicated with red arrows.

The sealer to dentin interface for the different sealers used is shown in Fig. 4. Micrographs are shown for each sealer type.

Irrespective of the irrigation protocol, all sealers penetrated into the dentinal tubules (marked with grey arrow in Fig. 4a). A rich dye infiltrated zone appeared at the tooth to sealer interface (marked with white arrow in Fig. 4a). This was more marked in the non-chelator groups (NaOCl and saline) for AH Plus. At HCSCs-dentin interface the dye infiltered zone was seen in Protocol A (NaOCl); Protocol B/D (NaOCl; NaOCl/chelator) for BioRoot; Protocol A (NaOCl/EDTA/NaOCl), Protocol D (NaOCl/EDTA-BC) and Protocol G (saline) for MTA Fillapex and Protocol B/D (NaOCl/chelator), Protocol F (EDTA-BC) and Protocol A/B (NaOCl/EDTA/NaOCl) for Totalfill.

AH Plus showed a good marginal adaption for irrigation protocols that included a chelator. The BioRoot and Totalfill also exhibited adequate marginal adaptation, except for Protocol A (NaOCl/EDTA) for the former, and Protocols A (NaOCl; NaOCl/EDTA) and F (EDTA-BC) for the latter. AH Plus displayed more material porosity than BioRoot and Totalfill (marked with blue arrow in Fig. 4a). MTA Fillapex displayed voids and cracks on its surface (marked with red arrow in Fig. 4a,b), with better interaction with dentin for Protocols B/D (NaOCl/chelator), E/F (chelators) and G (saline).

Irrigation protocols that contained chelator induced cracks (marked with white arrows) on root dentin when obturated with AH Plus and MTA Fillapex (Fig. 4b). The HCSCs exhibited poor adaptation in contact with the gutta-percha.

## Discussion

Effective root canal irrigation can only be achieved by using a combination of solutions^6,15^. The sequence and the chemistry of the irrigation solutions used establish the characteristics of the dentin surface and its subsequent interaction with the sealers employed in root canal obturation. In the current study, a number of commonly used endodontic irrigating solutions were tested individually and in combination to determine their effects on dentin in terms of microhardness, elasticity and also various methods of chemical analysis including elemental (EDS) and chemical (FT-IR) determination of the dentin constitution. Furthermore, surface microstructural analysis by SEM and topographic analysis by AFM were undertaken. The random distribution of the human teeth was performed to minimize variations in the mineral content of dentin that occurs with age caused by filling of the tubule lumens.

Sodium hypochlorite is used throughout the biomechanical preparation procedure during root canal therapy due its unique capacity to dissolve organic and necrotic tissues as pulp, bacterial and biofilm remnants^1,2^. It is capable of precipitating sodium chloride crystals inside the dentinal tubules and in the main root canal which may cause a bactericidal effect resulting from the loss of water content from the bacterial cells^16^. This precipitation has been observed in the current study. The concentration of sodium hypochlorite depends on the chlorine content it releases, which is positively correlated to its tissue-dissolving ability^17,18^. Chlorine is a strong oxidant that degrades long peptide chains of collagen^19^, resulting in N-chloroamines^20^ indicated by traces of chlorine on the surface of dentin observed in this study. Deproteination of dentin by NaOCl is not uniform. The smear layer created by the combined use of hand and rotary instruments has different amounts of organic mass^21^ which creates an irregular degradation pattern of superficial and sub-superficial encapsulated collagen.

In the structural architecture of dentin, the soft tissue collagen is encapsulated and protected by nanocrystalline carbonated apatite. Since NaOCl has a low molecular weight (74.4 Da) it spreads on the intrafibrillar water volume of apatite-encapsulated collagen matrix^22^. The contact surface of collagen with sodium hypochlorite leads to oxidative chemical degradation of collagen from the “superficial subsurface” of mineralized dentin, creating a collagen-depleted mineral ghost layer (Table 2) with a brittle structure^22,23^. In the current study, the mechanical properties of dentin are modified in 5 min of collagen degradation by the higher concentration (5%; Table 2; G4) of NaOCl. Protocol B and D show apparently conflicting results. The unpaired t-test that was performed comparing the differences between Initial and Group 4 among both protocols showed no significant differences indicating that the variations in the VHN values that occurred between the other groups within the protocol influenced the statistical analyses directly.

Advocated as an adjunct irrigant to NaOCl, the EDTA disodium salt removes the inorganic matter of the smear layer. Its reaction with the calcium ions in hydroxyapatite crystals can alter the mineral content of root dentin as verified in the current study^24^. Benzalkonium chloride is a surfactant that enhances the antimicrobial action of EDTA and its wettability on dentin. Despite the reduced surface tension of the mixture, there was no improvement in the ability of EDTA to remove the smear layer^25^, as shown in SEM images (Fig. 1a); or lead to a decrease in the microhardness of dentin, caused by the addition of a surfactant, as previously suggested^26^. When these chelators were used alone, they were less effective in widening the dentinal tubules and were more effective when used in conjunction with sodium hypochlorite^27^. The spectra obtained from FT-IR analysis showed that previous degradation of the organic portion of smear layer on the root canal surface by sodium hypochlorite led to further exposure of the inorganic matter, favoring the removal of Ca^2+^ by the chelators^28,29^. Although the irrigant sequence NaOCl and chelator led to the widening of the dentinal tubules, small amounts of smear layer remained on the dentin surface even after 3 min of flushing with the chelator. As smear layer removal is time dependent, the optimum working time of 1 min recommended for EDTA^4,30^ was not enough time for effectively cleaning the surface when it was used as a final flush. This is in accordance with the pilot study of this work that establish 3 min chelator working time to be more effective than 1 min.

The higher concentration of NaOCl followed by EDTA significantly changed the dissolution of inorganic matter and microhardness of dentin. These results strengthened the idea that subsurface damage caused by 5% NaOCl was higher, resulting in a collagen-depleted mineral ghost layer with unbound crystallites and non-uniform deproteinization channels^22^. The absence of statistical differences in the decrease in organic matter could be explained by the sub superficial measurements of FT-IR that penetrated to a depth of 1–2 μm^31^, masking the damages caused by NaOCl at a deeper level. EDTA exposed the underlying destruction caused by NaOCl, explaining the rougher topography on dentin surface with eroded dentinal tubules which was more pronounced than when using 2% NaOCl and EDTA or EDTA used on its own (Figs. 1a, 3). A three-dimensional structure with decalcified areas could be generated by the deep penetration of EDTA into the non-uniform channels and empty spaces caused by 5% NaOCl, creating a brittle structure. There was no significant reduction in microhardness and carbonate/phosphate ratios for dentin irrigated with 5% NaOCl followed by EDTA-BC, indicating a weaker chelating solution. All data suggests that the erosion of dentin was not exclusively induced by NaOCl or a chelator, but by the way the two were applied in an irrigation sequence. The modified dentin substrate after the irrigation sequence of NaOCl and EDTA will be susceptible to acids produced by acidogenic bacteria that remain in the root canal after obturation which may slowly dissolve the collagen-depleted apatite crystallites within the mineral layer creating clinical pathways for bacteria to enter^32^. A final rinse with NaOCl appeared to be beneficial for enhancing disinfection in the root canal and removing rests of organic debris^6,15^. The exposed and compromised fibrils were flushed away from the dentin surface, creating a flatter, smear layer-free surface. The peptide fragments and unbound crystallites on surface were easily dissolved resulting in enlarged tubular orifice diameters that were larger than those of the tubules^4,33,34^. At a subsurface level, the irregular dentin structure compromised by previous degradation of its organic and inorganic matter created faster pathways for spreading the NaOCl. As this occurred at a deeper level, peptide fragments and unbound crystallites, created by deproteinization caused by the final flush with NaOCl, may have had more difficulty with reaching the surface, which was morphologically observed as canal wall erosion in the intertubular dentin. The differences in the results observed for the Young’s modulus and Vickers microhardness can be attributed to the sensitivity of the test carried out and the area of analysis in the test being undertaken^31,35^. Nanoindentation measurements probed the molecular scale on the surface of the dentine. The decrease of Young’s modulus after treatment with saline point out the need of using untreated dentin as a control in AFM analysis in dentistry.

The current study investigated an epoxy resin-based sealer, a salicylate resin and MTA-based sealer and two hydraulic calcium silicate cements (HCSCs), assessing their interaction with the dentin modified by the various irrigation protocols and also their ability to change the dentin properties after application. AH Plus—an epoxy resin-based sealer—interacted with dentin through the sealer tags that penetrated into the dentinal tubules forming a mechanical^36^ and chemical bond. The latter occurs when the amino groups of the dentinal collagen bond to epoxy rings of AH Plus^13,37^. The proteolytic action of NaOCl on collagen makes chemical interaction poor^13^, enhancing the gaps at AH Plus-dentin interface. As there were organic and inorganic parts of smear layer present in the dentin irrigated with saline solution, a lower level of dentinal tubule penetration was shown. Chelators removed the inorganic smear layer, exposing dentin collagen fibrils, which enhanced the sealer penetration and better interaction of the sealer with the substrate. NaOCl combined with chelator groups improve the fluid-tight seal of the obturation^38,39^.

A failed collagen network impregnation by resin leads to material deterioration and loss of dentin bond strength over time^40^. MTA Fillapex, which is composed of a salycilate resin matrix filled with MTA, may prevent degradation of the water-rich resin-sparse collagen matrix by endogenous matrix metalloproteinases^41^, as water is progressively replaced by apatites. However, MTA Fillapex underwent shrinkage‐related stress during setting reaction due salicylate resin in its composition^42^. Unfilled spaces inside the MTA Fillapex mass and its detachment from dentinal walls led to gaps at the sealer-dentin interface^38,43^ also evident in the current study. A less defined dye infiltered zone was evident when MTA Fillapex was placed in contact with dentin prepared with NaOCl/chelator or EDTA.

Although the resin-based sealers rely on the chelation and widening of dentinal tubules with sealer tags to improve their adaptation^44^, the HCSC-sealers exhibited good material adaptation even when the dentine was not conditioned with chelators. HCSCs are calcium silicate based materials promoted for their bioactivity following it set. Totalfill is a premixed ready-to-use biphasic cement^45^ that set relies on biological fluids^46^. The moist environment on dentinal tubules may not reach it inner part inside root canals walls, as material dissolution was presented. The faster setting process of BioRoot relies on a water based liquid and a cloride accelarator^47^, thus explaining the higher amounts of chlorine on dentin for this sealer.

Following hydration, HCSCs dissolve, react, diffuse and precipitate as Ca(OH)_2_ and calcium silicate hydrate. The latter act as nucleation centres to produce surface apatite crystals, leading to the formation of a chemical bond along the sealer–dentin interface, but also within the collagen fibrils^48^. These chemical bonds seem to have a positive effect on reinforcing the remaining tooth structure^12^, proved by a recovery on dentin microhardness regardless of irrigation protocol following MTA Fillapex and Totalfill obturation. The lower viscosity when compared to BioRoot aids a deeper entrapment of the sealer within dentin through opened dentinal tubules^47,49,50^.

Leaching from HCSCs results in access to the water compartments of intrafibrilar apatite—paramount in maintaining dentin mechanical properties^51^—on mineralized (286 Da)^52^ and demineralizated collagen fibrils (6000 Da)^53^. Size exclusion characteristics of collagen was shown by tagging the sealers with water soluble tracers as Orange G (453.38 Da) under CLSM analysis. The extensive use of chelators (229.2 Da) may lead to dissolution of intrafibrillar apatite, which resonates with the lower levels of dentin Ca^2+^ following Protocol E and F. Fluorophores leaching from the HCSCs are supposed to migrate with the water through porosity of demineralizated collagen fibrils^53^, made up by tubules and intra/interfibrillar spaces on intertubular dentin, but may get entrap on the underlying layer of mineralised dentin whose porosity is composed only of tubules. This resulted in: a highly refletive band high above the dentin interface; collagen to collapse^54^ and dentin to crack in 2/24 samples.

Collagen caustic denaturing is followed by mineral diffusion, indicated by Ca-, Si- and fluorophores-rich areas leaching from HCSCs^9^ within the structure of dentin. This ion exchange layer called “mineral infiltration zone” (MIZ) has been indentified in previous studies using Rhodamine B (Sigma-Aldrich, St Louis, MO; 479.01 Da) and Fluorescein (Sigma-Aldrich, St Louis, MO; 332.30 Da) water-tracers dyes^9–11^. In a wet interface they follow fluid filtration as long as their molecular sizes are smaller than the slit width^55^. MIZ may correspond well with the transition zone between mineralizated collagen and water rich leakages on denature collagen, with biomineralization potencial. Porosity of peptide fragments and unbound crystallites stuck on the dentin walls increase intensity of fluorescent dye^56^ with a molecular-sieve effect on eroded dentin walls that may protect dentin from further aggression.

As shown in Table 4, all sealers enhanced calcium ions on dentin. Opposite to HCSCs, calcium is not readily soluble in water on AH Plus composition, being present as calcium tungstate^57^. Fluorophores may leach out from AH Plus and trace the smear layer moisture along dentin walls coated with non chelator groups due it powerful affinity for water-rich regions. These leaching effect of water-soluble tracers results on markers tracing the moisture of dentin instead of the sealer. Suggested as a HCSCs-marker substitute, Fluo-3 binds to the calcium in the sealer composition to emit fluorescence^58^. Nonetheless, the reduction of avaliable calcium may modify hydration kinetics, calcium-hydroxide release and thus, HCSCs properties.

CLSM showed ability of distinguishing sealer internal morphology, as it can slice the sealer in very thin optical sections. HCSCs showed a denser mass with lower incidence of porosity than AH Plus, also confirmed under nano-CT analysis in recvious studies^3^.

## Conclusions

The smear layer is removed more efficiantly by the consecutive use of NaOCl/EDTA/NaOCl. The use of sodium hypochlorite led to a reduction in dentin microhardness. This protocol resulted in an optimal sealer adaptation for all sealers but the dentin microhardness was not recovered to the original values for all HCSC sealers. The HCSC sealers did not rely on chelator irrigating solutions for optimal material adaptation to dentin at the sealer dentin interface unlike the AH Plus. The sealer interaction with the dentin results in a water-rich zone with biomineralization potenial and a molecular-sieve effect on eroded dentin walls, that protect dentin from further aggression.

## Methods

The following irrigating solutions were used:

- Sodium hypochlorite 2% and 5% (NaOCl-2, NaOCl-5; Cerkamed, Stalowa Wola, Poland).
- 17% EDTA (EDTA; Sigma, Stoinheim, Germany).
- 17% EDTA/1% Benzalkonium Chloride (EDTA-BC; Sigma, Stoinheim, Germany).
- 0.9% saline solution (Fisher Scientific, UK).

The EDTA was prepared by mixing 17 g of the powder (99,9% of purity) (Sigma, Stoinheim, Germany) in 50 mL of deionized water. Sodium hydroxide (Sigma, Stoinheim, Germany) was added to aid the solubility of the powder and pH was stabilized with hydrochloric acid, to 7.4. The solution was made up to 100 mL and pH buffered by adding 0.02 M of phosphate (Sigma, Stoinheim, Germany). For the preparation of EDTA-BC, 1.05 mL of BC (95% of purity) (Sigma, Stoinheim, Germany) was added to 99 mL of EDTA before the stabilization of pH and the addition of 0.02 M of phosphate. For the saline solution 0.904 g of NaCl powder (99,53% purity) (Fisher Scientific, UK) was mixed in 100 mL of deionized water, obtaining a 0.9% solution.

### Irrigation protocols

The solutions were tested individually and also in sequences constituting an irrigation protocol which included an antimicrobial and a calcium chelator. Saline was used as a control. The irrigation sequences shown in Table 1 while the details of how each solution was employed is shown in Table 5.

**Table 5:** The amount and velocity of the antimicrobials and chelators undertaken during the irrigation protocols.

Antimicrobial (NaOCl)	5 mL/each instrument (S1, SX, S2, F1, F2, F3) 1 mL/10 s flow rate
Chelator or chelator/antimicrobial (EDTA or EDTA-BC)	5 mL/3 min
Antimicrobial (NaOCl final irrigation)	5 mL/3 min

### Assessment of effect of irrigation protocol on dentin characteristics

#### Dentin preparation

Five hundred and fifty extracted human single rooted teeth were obtained from the University of Birmingham Dental School Tissue Bank. Ethical approval for the use of human teeth from the University of Birmingham ethical committee (ethical approval number: 14/EM/2811-BCHCDent397) was granted and all methods were carried out in accordance with relevant guidelines and regulations. All experimental protocols were approved by a named institutional committee. This study does not include subjects who are under 18 years. The teeth were embedded in auto polymerizing epoxy resin (Epoxyfix; Struers GmbH, Ballerup, Denmark) to enable sectioning of the root along its long axis using a hard tissue microtome (Isomet, Buhler, Lake Buff, USA). The resulting halves were then ground using progressively finer diamond discs (Stuers ApS, Ballerup, Denmark) and diamond-impregnating solutions (Stuers ApS, Ballerup, Denmark) on an automatic polishing machine (Buehler Phoenix Beta Grinder/Polisherm, Dusseldorf, Germany), finishing with a silicon suspension of 1 μm.

#### Microhardness testing

The measurement of the dentin microhardness was obtained by a Durascan 20 Vickers microhardness tester (Emco Test, Kuchl, Austria) at a magnification of × 40 employing 9.807 N load and a 15-s dwell time. The results were given by Vickers hardness number (VHN). After the initial measurement, the samples (*n* = 3) were submitted to the chemical irrigation protocols listed in Table 1 and employed as indicated in Table 2. After each step of the irrigation regime, five indentations were made along the middle third of dentin, starting close to the root canal area moving inwards and a mean value was obtained for each sample. The differences between treated and untreated (initial) VHN measurements of dentin were used to assess alterations in microhardness.

#### Dentin morphology and composition

##### Scanning electron microscopy (SEM) and energy dispersive spectroscopy (EDS)

Dentin specimens were prepared as detailed in the “Dentin preparation” section and were treated using the chemical irrigation protocols listed in Table 1 and employed as indicated in Table 5 after which they were dried in a vacuum desiccator, attached to aluminium stubs and sputter coated with a conductive layer of gold using a TK8842 Gold Target (Emitech Limited, Ashford, United Kington).

The dentin ultrastructure after each stage of the irrigation protocol was assessed by scanning electron microscopy (SEM; Zeiss MERLIN Field Emission SEM, Carl Zeiss NTS GmbH, Oberkochen, Germany) at × 2 K magnification. High magnification scanning electron micrographs of the relevant structures on dentin were also taken at × 3 K and × 4 K. The imaging was performed together with the Energy Dispersive Spectroscopy (EDS) analysis in which is mandatory to set the voltage at 20KV. Despite of trying to avoid changes in the accelerating voltage, specimens that presented a charging at 20KV had their voltage reduced to achieve a better image resolution. The photomicrographs acquired were assessed independently by three previously trained, calibrated and blinded evaluators. For Energy Dispersive Spectroscopy (EDS) analysis, the follow parameters were used, EHT = 20 kV, Iprobe = 1000 pA and WD = 8.5 mm for a 35° take off (elevation angle). The alterations of dentin surface by different irrigation protocols were measured by the monitoring changes in the calcium, phosphate and chlorine. Each dentin sample (*n* = *3*) was analysed in triplicate on distinct areas.

##### Fourier transform infrared spectroscopy (FT-IR)

FT-IR was used to determine compositional changes on human dentin (*n* = 5) after each irrigation protocol (Table 1). The mean of three separate acquisitions of spectra data was obtained for each sample using a Nicolet 6700 FT-IR machine (Thermo Scientific Instruments Corp., Madison, WI, USA) and Omnic 8 software suite (Thermo Scientific Instruments Corp.) within the mid‐IR spectrum (range 1600–750 cm^−1^) at a resolution of 0.482 cm^−1^ and 32 scans. After scanning, the baseline tracing was performed, and the areas under the infrared bands amide III (1298–1216 cm^−1^), phosphate (1170–780 cm^−1^) and carbonate (888–816 cm^−1^) were calculated by Microsoft Excel. Subsequently, the ratio of the amide III/phosphate was determined indicating the organic components of dentin. The inorganic components were calculated by the carbonate/phosphate band area ratios. Because of the interpositions of the bands of carbonate and phosphate, the latter was subtracted from the former to obtain the real value of the phosphate band.

##### Atomic force microscopy (AFM) analysis

A Dimension 3100 AFM (Bruker, Santa Barbara, CA, USA) was used with a TAP 150GD-G cantilever made from silicon with a gold reflex coating, resonant frequency of 150 kHz, and a nominal spring constant of 5 N/m (Budget Sensors, Bulgaria) for imaging the surface and nanoindentation to calculate the Young’s modulus of dentin^35^. After calibrating the spring constant and deflection sensitivity of the cantilever, force curves were measured by moving the tip into the dentin surface up to a constant force of 125 nN. (*n* = *3*) Each of the force curves were 100 nm apart in a 1 μm^2^ grid. The Young’s Modulus was calculated from the contact portion of the force curve using the Hertzian model 5 μm^2^ images were measured using tapping mode and were processed and analyzed using a commercial software NanoScope Analysis.

### Assessment of the dentin to sealer interface

The effect of the different sealers on the dentin after the different irrigation protocols outlined in Tables 1 and 5 was assessed by evaluating the microhardness of the dentin and also by assessing the dentin to sealer interface using SEM/EDS and confocal laser scanning microscope (CLSM).

The following sealers were investigated.

- AH Plus (Dentsply DeTrey GmbH, Konstanz, Germany);
- BioRoot (Septodont, Saint Maur‐des‐Fosses, France);
- MTA Fillapex (Angelus Dental Solutions, Londrina, SP, Brazil);
- Totalfill BC Sealer (FKG Dentaire, La-Chaux-de-Fonds, Switzerland).

#### Tooth preparation

The root canals of single rooted human teeth (*n* = 3) were instrumented using an in‐and‐out pecking motion of about 3 mm in amplitude with a light apical pressure to the working length (1 mm short of the apical foramen) with the ProTaper System (Dentsply Maillefer, Ballaigues, Switzerland) finishing with a master apical file size of an F3. With the aid of a plastic syringe and capillary tip cannula (Ultradent, South Lake City, USA) abundant irrigation was performed according to the irrigation protocol in Tables 1 and 5.

The sealers were prepared following the manufacturer’s recommendations, and they dispensed in the root canal using a syringe. Following obturation, the samples were stored in an oven at 37 °C for 7 days. The teeth were then embedded in auto polymerizing epoxy resin (Epoxyfix; Struers GmbH, Ballerup, Denmark), then sectioned longitudinally for SEM/EDS analysis and cross sectioned for CLSM using a hard tissue microtome and polished using an automatic polishing machine as described previously.

#### Microhardness testing

The microhardness of dentin in contact with the sealers for 7 days was assessed using the methodology outlined in the “Assessment of effect of irrigation protocol on dentin characteristics” section. This data was also compared to the microhardness of the dentin assessed in that section. This enabled the evaluation of the effect of the sealers on recovery of the dentin.

#### Assessment of the dentin to sealer interface

##### Scanning electron microscopy (SEM) and energy dispersive spectroscopy (EDS)

Energy dispersive spectroscopy in the scanning electron microscope (SEM/EDS) was performed on randomly selected areas on the dentin 50 µm away from the dentin-sealer interface.

##### Confocal laser scanning microscopy (CLSM)

To allow analysis under the CLSM, each sealer was mixed with Orange G dye (16230 Sigma-Aldrich, Dorset, UK) used in 0.1% concentration (*n* = 3). After tooth preparation, the samples were placed in a 35 mm petri dish, covered with water and examined with an inverted Leica TCS-SP8 confocal system equipped with an DM6 upright microscope and a 40x/0.80 HCX water immersion lens (Leica Microsystems GmbH, Mannheim, Baden-Württemberg, Germany) with an excitation/emission wavelength of 494/521 nm. Four randomly selected images were made *per* sample. The photomicrographs acquired were assessed independently by three previously trained, calibrated and blinded evaluators.

### Statistical analysis

The Kolmogorov–Smirnov test was used to verify the normality of data from all analyses. The values of dentin microhardness (dentin without sealer) and organic/inorganic matter ratios (Fourier Transform Infrared Spectroscopy) were normally distributed and submitted to one-way repeated measures analysis of variance (ANOVA) and Tukey’s multiple-comparison test to detect differences in the composition of the dentin among groups inserted on the same irrigant protocol. Absence of normality was observed from the data of dentin microhardness (obturated dentin), mineral content (Energy Dispersive Spectroscopy) and Young`s modulus (Atomic Force Microscope). Therefore, statistical comparisons between the groups were made by Kruskal–Wallis and Dunn test. All hypotheses tests were performed at a 95% confidence level. The unpaired t-test was performed for intra-group comparisons in the hardness measurements.
